# Bioactive Protein and Peptide Release from a Mucoadhesive Electrospun Membrane

**DOI:** 10.1007/s44174-023-00098-5

**Published:** 2023-06-21

**Authors:** Jake G. Edmans, Craig Murdoch, Paul V. Hatton, Lars Siim Madsen, Martin E. Santocildes-Romero, Sebastian G. Spain, Helen E. Colley

**Affiliations:** 1 School of Clinical Dentistry, University of Sheffield, 19 Claremont Crescent, Sheffield, S10 2TA UK; 2 Department of Chemistry, University of Sheffield, Brook Hill, Sheffield, S3 7HF UK; 3 AFYX Therapeutics, Ole Maaløes Vej 3, 2200 Copenhagen, Denmark

**Keywords:** Electrospinning, Drug delivery, Peptides, Oral mucosa, Mucoadhesion, Bradykinin, Insulin

## Abstract

Protein-based biologics constitute a rapidly expanding category of therapeutic agents with high target specificity. Their clinical use has dramatically increased in recent years, but administration is largely via injection. Drug delivery across the oral mucosa is a promising alternative to injections, in order to avoid the gastrointestinal tract and first-pass metabolism. Current drug delivery formulations include liquid sprays, mucoadhesive tablets and films, which lack dose control in the presence of salivary flow. To address this, electrospun membranes that adhere tightly to the oral mucosa and release drugs locally have been developed. Here, we investigated the suitability of these mucoadhesive membranes for peptide or protein release. Bradykinin (0.1%) or insulin (1, 3, and 5%) were incorporated by electrospinning from ethanol/water mixtures. Immersion of membranes in buffer resulted in the rapid release of bradykinin, with a maximal release of 70 ± 12% reached after 1 h. In contrast, insulin was liberated more slowly, with 88 ± 11, 69.0 ± 5.4, and 63.9 ± 9.0% cumulative release of the total encapsulated dose after 8 h for membranes containing 1, 3, and 5% w/w insulin, respectively. Membrane–eluted bradykinin retained pharmacological activity by inducing rapid intracellular calcium release upon binding to its cell surface receptor on oral fibroblasts, when examined by flow cytometry. To quantify further, time-lapse confocal microscopy revealed that membrane–eluted bradykinin caused a 1.58 ± 0.16 fold-change in intracellular calcium fluorescence after 10 s compared to bradykinin solution (2.13 ± 0.21), relative to placebo. In conclusion, these data show that electrospun membranes may be highly effective vehicles for site-specific administration of biotherapeutic proteins or peptides directly to the oral mucosa for either local or systemic drug delivery applications.

## Introduction

Peptide and protein biologics are a rapidly growing class of pharmaceuticals with high clinical success rates, high specificity, high potency and low toxicity [1]. However, these types of therapeutics suffer from low oral bioavailability due to limited gastrointestinal absorption, enzymatic degradation and first-pass hepatic metabolism, and are therefore almost exclusively administered parenterally by injection. This has significant associated drawbacks including patient discomfort, non-compliance due to trypanophobia and, in many cases, the need for administration by healthcare professionals. For these reasons, there is much interest in drug delivery via mucosal surfaces [2, 3]. Transmucosal drug delivery via the oral cavity enables easy self-administration and is painless, thus increasing compliance rates. Furthermore, the ability to deliver biologics topically to the oral mucosa may enable new treatments for oral diseases whilst mitigating off-target side effects. Although there is much interest in this mode of delivery, progress is limited by the lack of suitable dosage forms that allow site-specific delivery to mucosal surfaces.

The only formulation that has successfully reached the market for transmucosal peptide delivery to date is the Oral-Lyn™ insulin aerosol spray for the management of type 1 diabetes [4]. Unlike competing alternatives, this formulation is absorbed through the buccal oral mucosa rather than the respiratory tract and uses a mixture of surfactants to enhance epithelial permeation as well as to allow aerosol formation [5]. The relatively high doses of insulin required and its poor epithelial permeability make it a challenging candidate drug for transmucosal delivery. Indeed, significant disadvantages of buccal insulin sprays include modest bioavailability of approximately 10% and the large volume of spray required to achieve a therapeutic dose [4]. In vivo investigations into transmucosal biologic delivery suggest that several hydrophilic lower molecular weight therapeutic peptides including glucagon-like-peptide 1 [6], salmon calcitonin [7], and desmopressin [8] delivered to the buccal or sublingual mucosa can permeate sufficiently to achieve therapeutically relevant systemic doses without the need for permeation enhancing excipients. Low molecular weight peptides with fewer rotatable bonds tend to permeate the oral epithelium more easily, facilitating uptake. Absorption of these molecules is often hypothesised to occur by passive diffusion through the epithelial paracellular spaces [9]. There is great potential to develop new dosage forms for the delivery of permeable proteins or peptides into or across the oral mucosa depending on epithelial or systemic targeting. However, previous studies have examined dosage forms with limited suitability for further translation, typically consisting of solutions or dissolving tablets/films that are readily removed from the mucosal surface by saliva, resulting in limited drug/tissue contact times [10, 11].

Electrospinning has recently attracted interest for the fabrication of oromucosal dosage forms with improved flexibility and high surfaces areas to facilitate drug release and mucoadhesive interactions [12, 13]. Furthermore, dual-layer electrospun membranes with hydrophobic backing layers are highly effective at promoting the retention of drugs on the oral mucosa in the presence of saliva flow [14, 15]. Previous studies investigating electrospinning for protein or peptide encapsulation have primarily focussed on emulsion and coaxial electrospinning, due to the perception that organic electrospinning solvents such as ethanol would cause protein denaturation during uniaxial electrospinning [16]. Alternatively, protein-loaded fibres have been prepared from aqueous solutions to avoid potential solvent-induced denaturation using polymers such as chitosan, silk or alginate [17–19]. We have previously developed a dual-layer mucoadhesive electrospun drug delivery patch consisting of a mucoadhesive inner membrane layer fabricated using Poly(vinylpyrrolidone) (PVP) and Eudragit® RS100, and a protective polycaprolactone-based outer membrane layer to facilitate site-specific delivery to the buccal mucosa [20]. These membranes were highly effective in phase II clinical trials in the delivery of clobetasol-17-propionate for treatment of erosive oral lichen planus [21], and have potential for several applications in oral medicine [13, 15, 22].

At present, only a few peptides with therapeutic potential have been incorporated into polymer fibres using solvent-based uniaxial blend electrospinning. Moreover, their pharmacological activity following elution from the polymer fibres has not been investigated [16, 23, 24]. In this study, bradykinin (a nine amino acid, 1060 Da peptide) or insulin (a 5.8 kDa protein) were incorporated into a novel mucoadhesive electrospun fibre membrane and their release kinetics examined to further investigate the versatility of the technology for mucosal delivery. Moreover, the biological activity of bradykinin following elution was assessed to determine if released molecules retain their biological activity.

## Materials and Methods

### Materials

Poly(vinylpyrrolidone) (PVP; MW 2000 kDa) and Eudragit® RS100 (RS100; MW 38 kDa) were kindly donated by BASF, Cheadle Hulme, UK and Evonik Industries AG, Essen, Germany, respectively. Bradykinin ELISA kit was purchased from Abcam, Cambridge, UK. Fluo-4 Direct™ calcium assay kit was purchased from ThermoFisher Scientific, Loughborough, UK. Recombinant bradykinin, insulin, and cell culture reagents were purchased from Sigma-Aldrich, Poole, UK.

### Electrospinning System

Electrospun membranes were fabricated using a system composed of a PHD2000 syringe pump (Harvard Apparatus, Cambridge, UK) and an Alpha IV Brandenburg power source (Brandenburg, UK). Plastic syringes (1 mL volume; Henke Sass Wolf, Tuttlingen, Germany) were used to drive the solutions into a 20-gauge blunt metallic needle (Fisnar Europe, Glasgow, UK). Electrospinning was performed at room temperature with a potential difference of 19 kV, a flow rate of 2 mL/h, and a flight path of 14 cm.

### Fabrication of Electrospun Membranes Containing Bradykinin or Insulin

Electrospinning solutions for bradykinin contained 0.1025 g/mL PVP and 0.1225 g/mL Eudragit® RS100 (by total solvent volume before mixing). PVP and RS100 were added to ethanol and mixed at room temperature using a magnetic stirrer until dissolved. Bradykinin was dissolved in ice cold phosphate-buffered saline (PBS), added to the polymer solution shortly before electrospinning and stirred until uniformly distributed, contributing 3% v/v to the final solvent composition. Placebo solutions and membranes were prepared using 3% v/v distilled water instead of bradykinin in PBS. For insulin, all electrospinning solutions contained 0.0825 ± 0.00125 g/mL PVP and 0.1025 ± 0.00125 g/mL Eudragit® RS100 (by total solvent volume before mixing). The required amounts of PVP and RS100 were added to ethanol and mixed at room temperature using a magnetic stirrer until dissolved. The required concentration of insulin was prepared in 2% acetic acid in PBS in a glass vial and vortexed for approximately 60 s with gentle heating until a clear solution was produced. Insulin solutions were combined with the polymer solutions at 20% v/v and mixed until homogenous shortly before electrospinning. Placebo solutions and membranes without recombinant peptides/proteins were prepared under identical conditions.

### Surface pH

Membrane samples (10 ± 0.5 mg) were placed in wells of a 24-well plate, submerged in 1 mL deionised water, and shaken briefly until wetted. After 10 minutes, the pH was measured using a pH meter (Hanna Instruments, Rhode Island, USA).

### Scanning Electron Microscopy

Electrospun membranes were imaged using a TESCAN Vega3 scanning electron microscope (SEM; Tescan, Cambridge, UK). Samples were sputter coated with gold and imaged using an emission voltage of 10 kV. All images were processed and fibre diameters measured using ImageJ software tools [25], using randomly generated coordinates and a superimposed grid to select fibres. Three images were analysed for each membrane composition with at least 10 measurements per image.

### Release Profile of Bradykinin from Electrospun Membranes

Samples of electrospun membranes (20 mg) containing bradykinin were immersed in 4 mL PBS and 10 μL samples taken at time intervals up to 4 h following vortexing for 5 s. Bradykinin concentrations were quantified using a competitive ELISA kit according to the manufacturer’s instructions (abcam, Cambridge, UK). To calculate percentage cumulative release, the active concentration was normalised against the theoretical maximum concentration based on the dry mass fraction of bradykinin in the electrospinning solution.

### Release Profile of Insulin from Electrospun Membranes

Membrane samples (3–6 mg) containing insulin were cut using a circular punch and placed in wells of a 12-well plate. Pre-warmed PBS (2 mL) was added to the wells and the samples incubated at 37 °C with shaking. 50 μL aliquots were taken every 0.5 h for 8 h and the volume replaced with fresh media. Protein concentration was measured using a Pierce™ bicinchoninic acid (BCA) assay kit as directed (ThermoFisher Scientific). Percentage cumulative release was calculated by normalising against the theoretical maximum concentration, based on the dry mass fraction of insulin in the electrospinning solution.

### Cell Isolation and Culture

Normal oral fibroblasts (NOF) were isolated from the connective tissue of biopsies obtained from the oral mucosa of patients during routine dental procedures with written informed consent (Ethical Approval No. 09/H1308/66) and conducted in accordance with the principles embodied in the Declaration of Helsinki. NOF were cultured in Dulbecco’s Modified Eagle’s Medium supplemented with foetal bovine serum (FBS; 10% v/v), 2 mM L–glutamine, 100 IU/mL penicillin and 100 μg/mL streptomycin [26].

### Intracellular Calcium Mobilisation Using Flow Cytometry

NOF were removed from cell culture flasks and resuspended in buffered Fluo-4 cell-permeant acetoxymethyl ester (ThermoFisher Scientific) at a suspension of 10^6^ cells/mL and incubated for 45 minutes at 37 °C followed by 30 minutes at room temperature. Bradykinin-loaded or placebo membranes (10 mg) were eluted in 2 mL PBS for 24 h and stored at − 20 °C prior to the experiment. A stock solution of bradykinin in PBS matching the theoretical concentration of the membrane eluent (5 μg/mL) was prepared as a positive control and stored in identical conditions. Samples and standards were diluted by a factor of 10 in PBS before assaying by flow cytometry. To determine intracellular calcium responses, fluorescence was measured at 488 nm excitation and 530 nm emission using a FACSCalibur flow cytometer (BD Biosciences, Wokingham, UK). For each sample, baseline fluorescence was measured for 40 s before sample addition (10 μL in 1 mL) after which fluorescence was measured for approximately 160 s. Change in relative fluorescent units (ΔRFU) was calculated by subtracting the median relative fluorescent unit value of the baseline from the maximum median fluorescence (over a 1 s interval) following sample addition. The experiment was performed in triplicate using three independently prepared sets of membranes and different batches of cells.

### Intracellular Calcium Mobilisation Using Confocal Microscopy

NOF (2.5 × 10^4^ in 25 µL) were seeded in a half-area glass-bottom 96-well plate and incubated overnight to give a confluent monolayer. Bradykinin samples and controls were prepared as previously described and diluted by a factor of 1000 prior to assaying. Before imaging, 25 µL buffered Fluo-4 cell-permeant acetoxymethyl ester was added and the cells incubated for 45 minutes at 37 °C, followed by 30 minutes at room temperature. Imaging was performed using a Nikon A1 laser scanning confocal microscope with 457–514 nm argon laser. Cells were imaged at room temperature with approximately 1200 cells per image to obtain a baseline. Samples (50 µL) were added, and images immediately acquired every 10 s for 60 s using identical capture settings. The fold-change in fluorescence was calculated from the mean intensity of each the micrograph relative to the baseline. All images were processed using ImageJ software tools using automatic brightness/contrast adjustment on each series of images. The experiment was performed three times using three independently prepared sets of membranes and different batches of cells.

### Data Analysis

All data and statistical analyses were performed using GraphPad Prism 9.4.1 software (GraphPad Software, La Jolla, USA). Student’s *t*-test was used for pairwise comparisons whilst one-way ANOVA with Tukey’s post hoc test was used to compare differences between groups and results considered statistically significant if *p* < 0.05.

## Results

### Incorporation of Bradykinin or Insulin into Electrospun Membranes

**Fig. 1:**
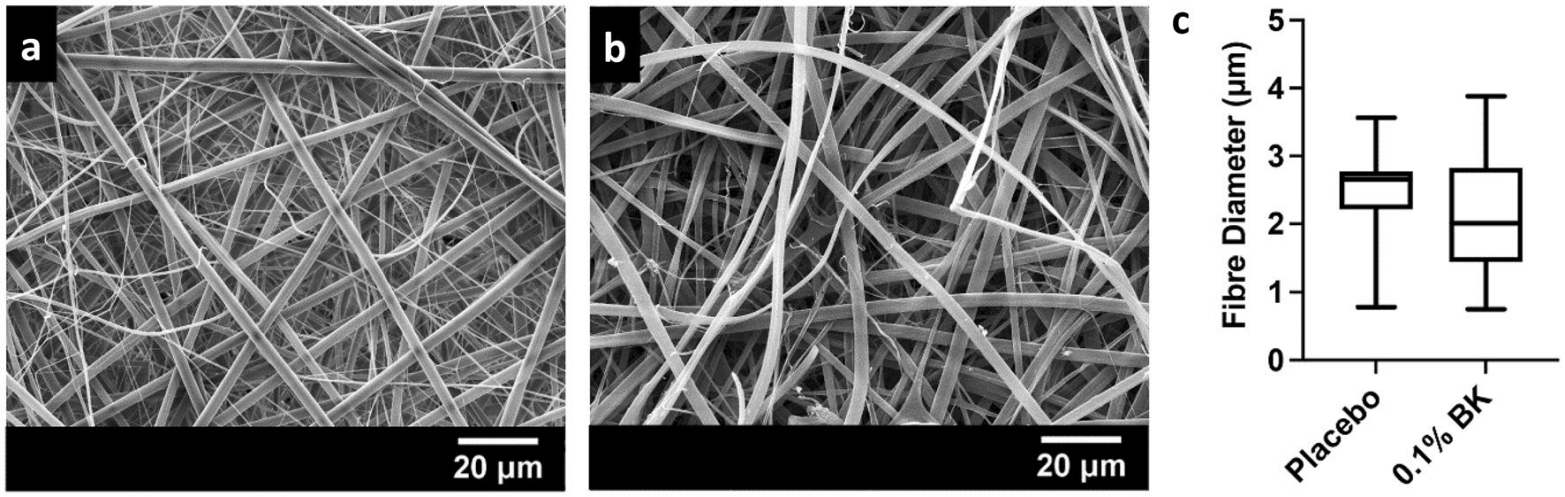
Characterisation of bradykinin-loaded mucoadhesive electrospun membranes. Bradykinin was incorporated into polymer fibres by mixing with ethanolic polymer solutions immediately before electrospinning and the resulting fibres imaged by SEM. SEM micrographs of **a** control electrospun fibres **b** electrospun fibres containing 0.1% w/w bradykinin (BK), scale bar = 20 µm. **c** Fibre diameter distribution presented as box and whisker plots displaying median, interquartile range and range (*n* = 30). Statistical analysis was performed by Student’s *t*-test

Electrospun membranes containing 0.1% w/w bradykinin by dry mass were prepared by electrospinning from a solution of 97% v/v ethanol. SEM images revealed a randomly aligned fibre morphology, with few defects, and an average diameter of 2.23 ± 0.81 μm that was similar to control membranes (Fig. 1).

**Fig. 2:**
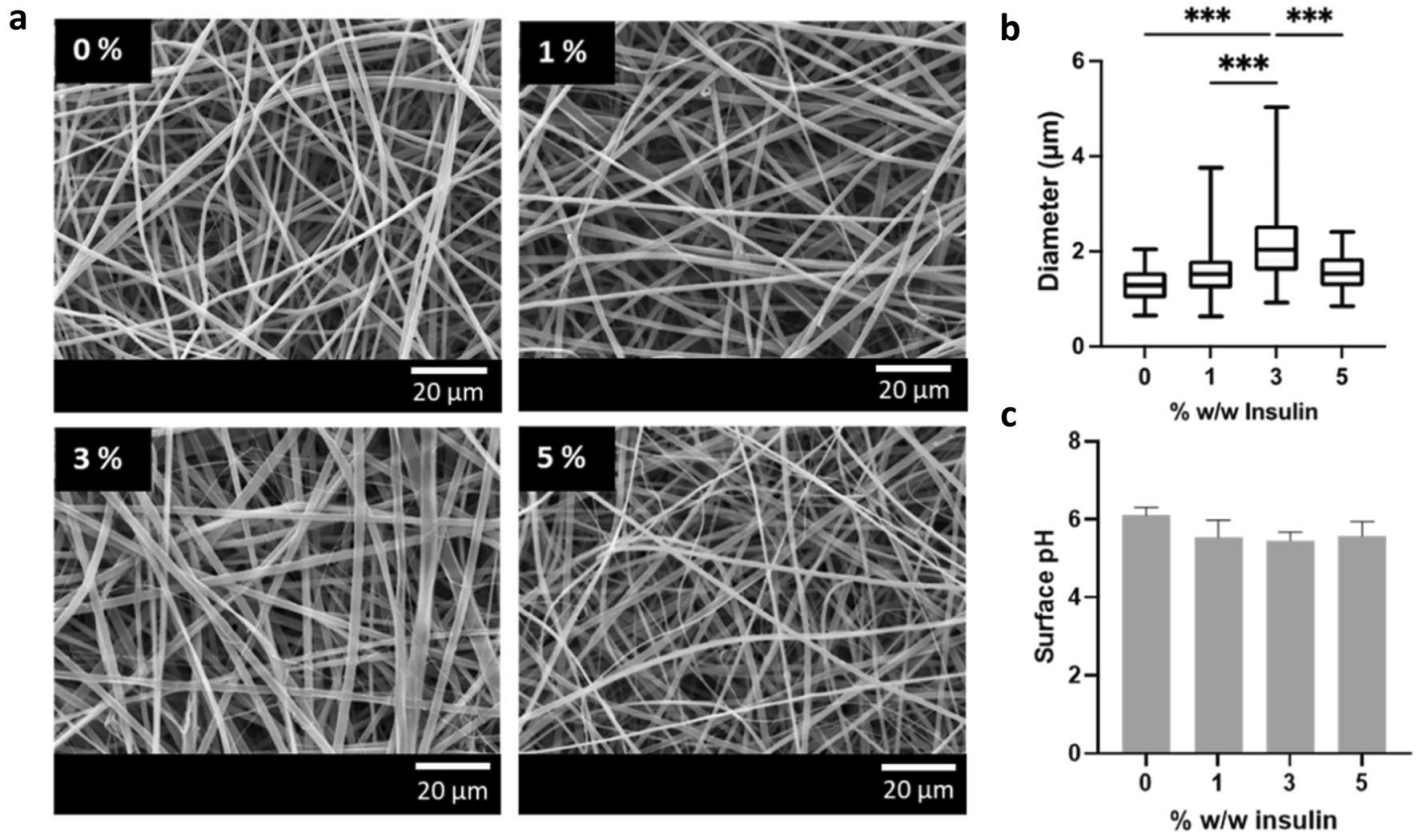
Preparation of mucoadhesive electrospun fibres containing 0, 1, 3, 5% w/w insulin. Insulin was dissolved in 2% v/v acetic acid in PBS and mixed 1:4 v/v with an ethanolic polymer solution shortly before electrospinning. **a** SEM micrographs of electrospun fibres containing 0, 1 3, and 5% w/w insulin. **b** Fibre diameter distributions presented as median, interquartile range, and range. Data were analysed using one-way ANOVA with Tukey post hoc test. ***p < 0.001 (*n* = 30). **c** Surface pH of electrospun fibres presented as mean ± SD (*n* = 3) and analysed using one-way ANOVA with Tukey’s post hoc test

Due to the relatively low aqueous solubility of insulin, a solvent composition of 80% v/v ethanol was used to allow the mixing of a larger volume of aqueous protein solution. The aqueous component of the electrospinning solution was acidified with acetic acid to dissolve the insulin and membranes were fabricated to contain 0, 1, 3, and 5% w/w insulin by dry mass. SEM analysis revealed a defect-free fibrous morphology in the absence or presence of insulin (Fig. 2a). Membranes without insulin had a mean fibre diameter of 1.29 ± 0.36 μm. Inclusion of insulin at 3% w/w or 5% w/w resulted in significantly increased diameters of 2.16 ± 0.78 μm and 1.55 ± 0.39 μm, respectively (*p* < 0.001; Fig. 2b).

To investigate the effect of the use of acetic acid on surface pH, membranes were immersed in a small volume of distilled water before measuring pH with a digital probe. All samples prepared using this method had a surface pH of approximately 6 (Fig. 2c), whereas previously fibre formulation without the use of acetic acid had a pH of 8.2 [15].

### Release of Bradykinin and Insulin from Electrospun Membranes

**Fig. 3:**
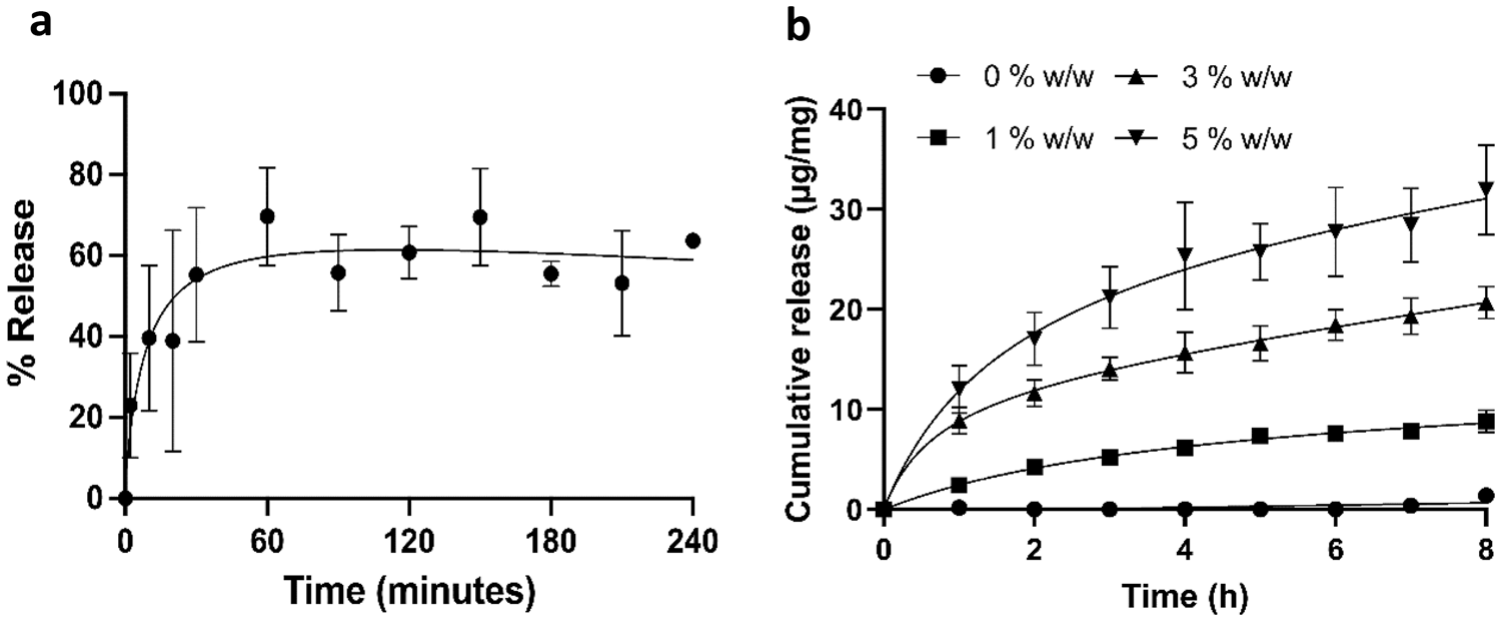
Release of bradykinin and insulin from mucoadhesive membranes following immersion in PBS. Electrospun membrane samples were eluted in PBS at 37 °C. Bradykinin concentrations in the eluted PBS were measured by ELISA whilst insulin release was measured by BCA assay. **a** Percentage release of bradykinin over 240 minutes **b** cumulative insulin release per mg of membrane over 8 h. Data are presented as mean ± SD (*n* = 3). Statistical analysis for insulin was performed at the 8 h timepoint by one-way ANOVA with Tukey’s post hoc test

The release profile of bradykinin and insulin from membranes was measured by eluting in PBS and measuring bradykinin concentration by ELISA and insulin by BCA protein assay. Bradykinin release was rapid, with 55 ± 17% of the theoretical total dose released within 30 minutes. Maximum bradykinin release of 70 ± 12% was observed after 1 h (Fig. 3a). Release of insulin was slower than that of bradykinin, with maximal but incomplete release after 8 h (Fig. 3b). When evaluating liberation of insulin per mg membrane, release was dependent on insulin content, with the 5 and 3% w/w patch releasing significantly more insulin than the 1% w/w (*p* < 0.001 and *p* < 0.01, respectively) and placebo membrane (*p* < 0.001) (Fig. 3b). The percent cumulative insulin release after 8 h corresponded to 88 ± 11, 69.0 ± 5.4, and 63.9 ± 9.0% of the total encapsulated dose for membranes containing 1, 3, and 5% w/w insulin, respectively.

### Membrane–eluted Bradykinin Stimulates Intracellular Calcium Release in Normal Oral Fibroblasts

**Fig. 4:**
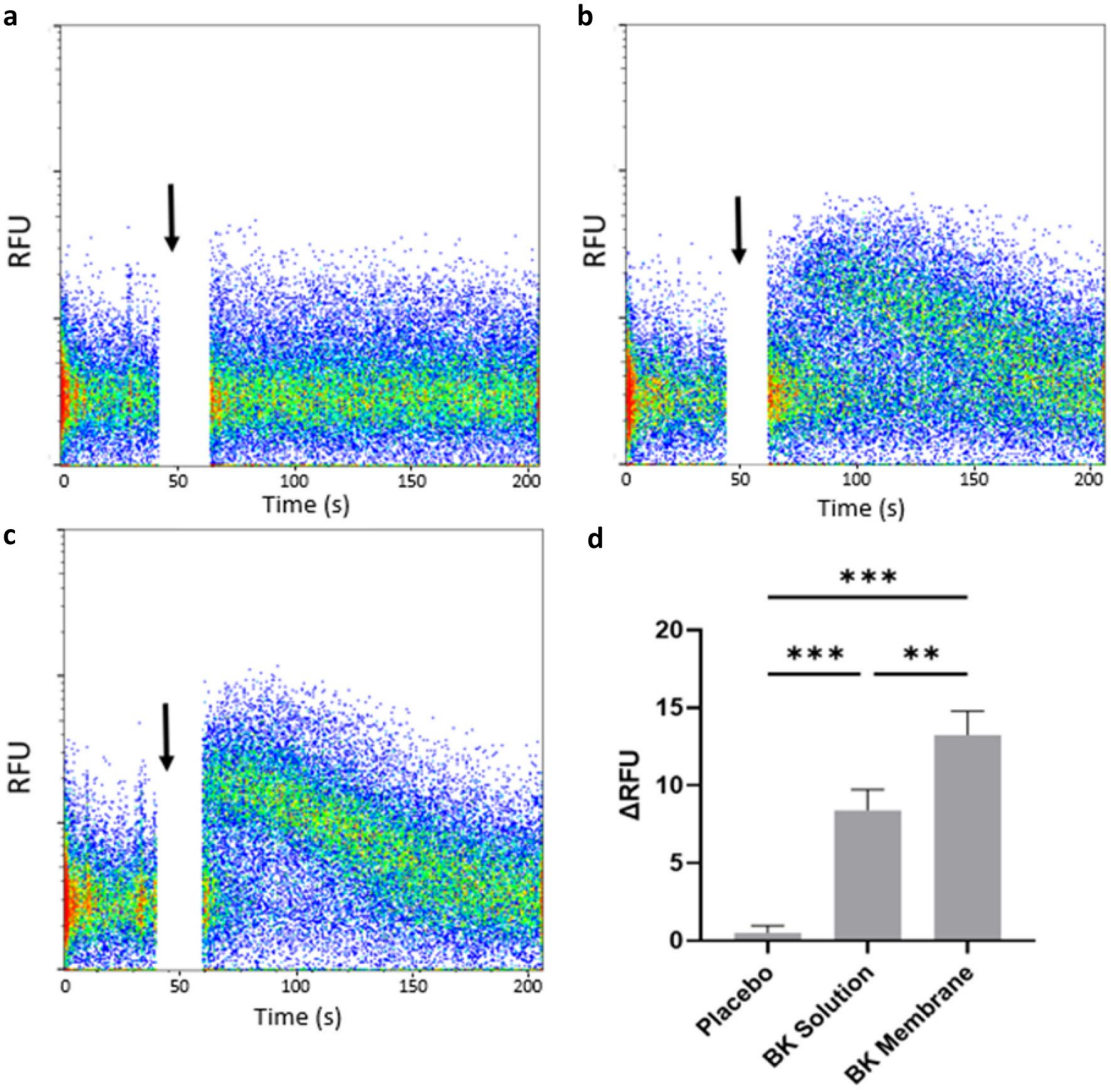
Bradykinin functionality was preserved following release from electrospun mucoadhesive membranes. Intracellular calcium mobilisation in primary fibroblasts upon stimulation was determined over time using flow cytometry. Each dot represents a fluorescence measurement for an individual cell. Baseline fluorescence was acquired for 40 s before addition of samples (black arrow). **a** Placebo membrane eluent **b** recombinant bradykinin (BK) solution **c** BK electrospun membrane eluent **d** change in relative fluorescence units (RFU) were calculated by subtracting baseline median fluorescence from the maximal median fluorescence following sample addition. Data is presented as mean ± SD (*n* = 3) and analysed using one-way ANOVA with Tukey’s post hoc test. **p < 0.01, ***p < 0.001

The release of insulin from other polymer-based drug carriers for diabetic therapy has previously been reported in several studies [4, 17, 27–29]. Therefore, we decided to focus further experiments on bradykinin as a novel exemplar test molecule for membrane release. Bradykinin is a hydrophilic nine amino acid (1060 Da) peptide that exerts its effects on cells such as fibroblasts, smooth muscle, and endothelial cells that form blood vessels, causing vasodilation and increasing vascular permeability following engagement of G-protein coupled bradykinin receptors. To test whether the biological activity of bradykinin was preserved following release from membranes, its effect on intracellular calcium mobilisation following binding to its cell surface G-protein coupled receptor expressed by NOF was measured using flow cytometry. Bradykinin-containing membranes were eluted in PBS for 24 h and the supernatant added to cell suspensions at 5 ng/mL (assuming 100% loading efficiency and complete release from the membranes) to approximate physiological concentrations. The addition of placebo membrane eluent to NOF had negligible effect on fluorescence as a readout for intracellular calcium release, causing a 0.51 ± 0.46 maximum change in relative fluorescence units (RFU) compared to baseline (Fig. 4a and d). Recombinant bradykinin solution caused a maximum 8.4 ± 1.3 ΔRFU (Fig. 4b and d) whilst electrospun membrane–eluted bradykinin caused a 13.2 ± 1.5 maximum ΔRFU (Fig. 4c and d).

**Fig. 5:**
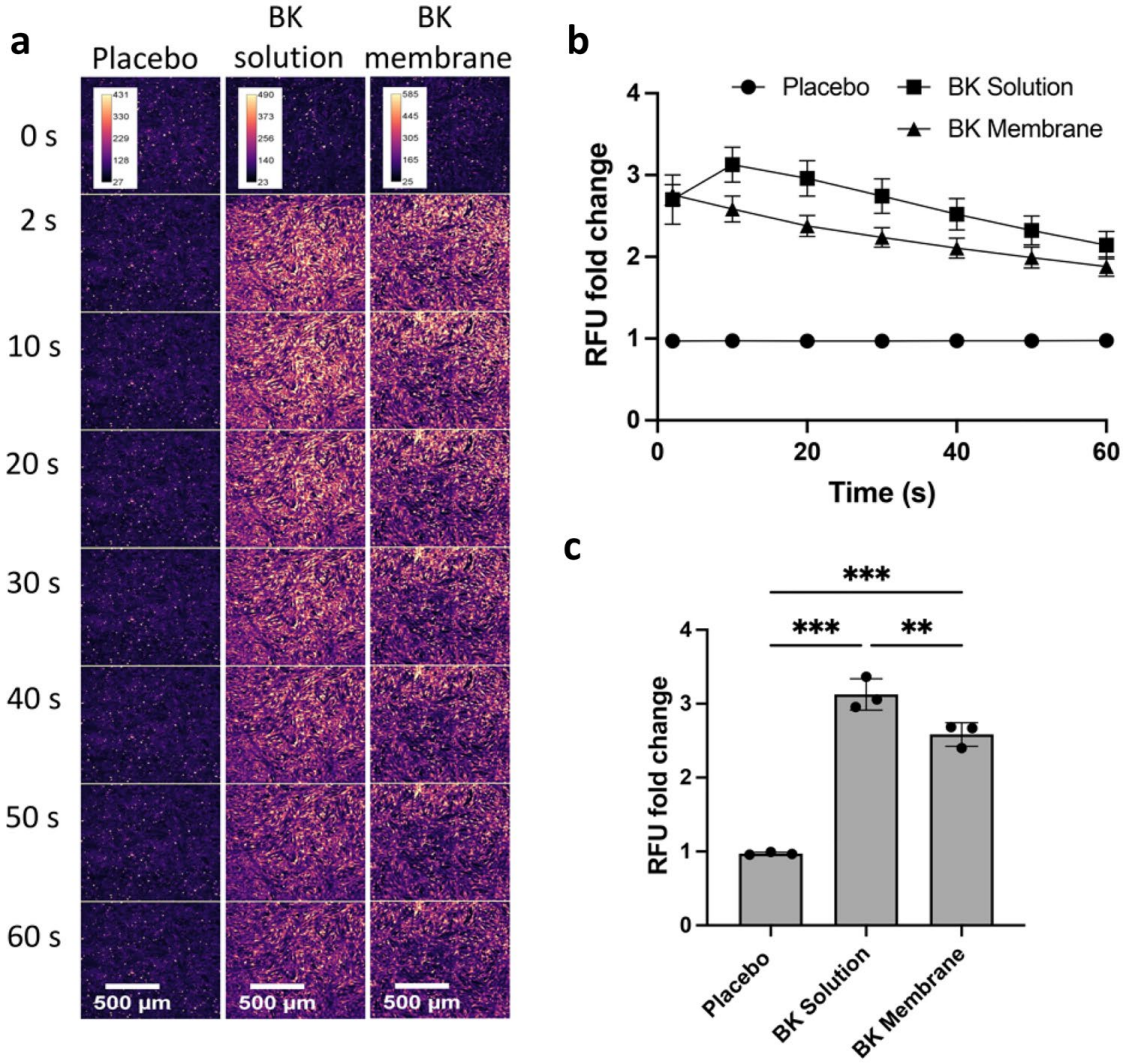
Membrane–eluted bradykinin increases intracellular calcium mobilisation in oral fibroblast monolayers. **a** Representative series of confocal micrographs showing the change in calcium mobilisation over time in primary fibroblast monolayers following treatment with PBS, placebo membrane eluent, bradykinin (BK) solution (2.5 ng/ml), or BK membrane eluent, scale bar = 500 μm. **b** Fold-change in fluorescence relative units (RFU) over time following addition of solutions. **c** Fold-change in RFU after 10 s for each solution. Data is presented as mean ± SD (*n* = 3). Statistical analysis was performed using one-way ANOVA with Tukey’s post hoc test. **p < 0.01, ***p < 0.001

The effect on intracellular calcium mobilisation was also observed in NOF monolayers by confocal microscopy. Bradykinin samples were added at 2.5 ng/mL (assuming 100% loading efficiency and complete release), after which fluorescence micrographs were periodically captured for up to 60 s (Fig. 5). An eluted placebo membrane was used as a negative control and as expected, produced negligible change in fluoresce. Bradykinin in solution and as elute from the membrane cause a rapid (within 2 s) increase in intracellular calcium (Fig. 5a). Bradykinin in solution caused a 2.13 ± 0.21 fold-change in fluorescence intensity peaking at 10 s (*p* < 0.001) relative to placebo, after which fluorescence intensity decreased linearly over time (Fig. 5b and c). Membrane–eluted bradykinin produced similar behaviour, peaking at approximately 2 s, causing a 1.58 ± 0.16 fold-change after 10 s (*p* < 0.001) relative to placebo (Fig. 5b and c).

## Discussion

Electrospun mucoadhesive membranes have been developed to address the lack of formulations that enable site-specific drug delivery to the oral mucosa [12]. These membranes have been successfully developed for the delivery of several small molecule drugs [13, 15, 22, 30–32], lysozyme [33], a robust antimicrobial enzyme, and antibody fragments [34], suggesting suitability for oromucosal protein delivery. In order to determine the potential of electrospun membranes for transmucosal peptide delivery it is necessary to demonstrate that a peptide can be incorporated and released at a suitable rate whilst maintaining pharmacological activity. Bradykinin is a small but highly potent vasoactive peptide. It was used in this study as an exemplar test molecule to provide proof-of-concept data that other such small peptides could be incorporated into electrospun membranes, released and retain their biological activity.

Drug delivery from a membrane or patch applied to the oral mucosa is time-limited due to the requirement of patients to refrain from eating and drinking upon membrane application. A previous study observed a clinically measured membrane residence time of approximately 100 min for similar membranes applied to the human buccal mucosa [15]. Therefore, efficient peptide release over a 2 h period would be ideal for delivery within this timeframe. Indeed, maximal bradykinin release corresponding to around 70% of the total dose was detected after just 1 h. This rapid release is consistent with a release mechanism controlled by the swelling of highly hydrophilic electrospun fibres [35]. The release profile is similar to those previously reported for hydrophilic proteins encapsulated within swelling electrospun fibres where it was found that lysozyme was rapidly eluted from an identical polymer system, with approximately 90% of the dose released within 1 h [33]. In addition, Stie et al., showed that mucoadhesive fibres consisting of blended poly(ethylene oxide) and chitosan rapidly released the highly soluble peptide desmopressin through a similar mechanism [14]. Here, desmopressin release was even faster than observed in our system, reaching 80% release within 20 minutes. This can be attributed to narrower fibre diameter of approximately 200 nm, leading to a higher surface area and faster fibre swelling.

Insulin was selected as a second model peptide to investigate the versatility of the patches for the delivery of larger, less soluble peptides, with the potential membrane application for sustained insulin delivery for diabetics. Insulin has low aqueous solubility at neutral pH. However, it is moderately soluble at pH 3, and if required, the pH of the solution can be adjusted back to pH 7 without causing protein precipitation [36]. The solubility is further increased by increasing the ionic strength of the solution, for example by using acidified PBS instead of acid in deionised water. Relatively high therapeutic doses in the order of hundreds of micrograms several times daily are required for managing diabetes [37]. Therefore, to achieve suitably high loading in the mucoadhesive layer of the patch, the aqueous portion of the electrospinning solvent was increased to 2% v/v. Acidified PBS was used to solubilise insulin before mixing with the ethanolic polymer solution. This method allowed the fabrication of defect-free fibres containing 0, 1, 3, 5% w/w insulin.

The diameter of fibres containing 1% w/w insulin had a narrower diameter than that those containing bradykinin. This is likely a result of the increased conductivity of the electrospinning solution, due to the higher water and salt content, which produces more rapid fibre elongation in an electric field. The fibres were also narrower than those previously observed for fibres containing 1% w/w lysozyme, also prepared with 80% v/v ethanol [33]. This is likely because PBS was used exclusively instead of deionised water, resulting in a more conductive solution, which is associated with narrower fibre widths [38]. Fibres containing 3% w/w insulin were significantly wider than those with 1 or 5% w/w. Insulin concentration affects both solution conductivity and viscosity that may affect fibre diameter. The diameter of electrospun fibres can be modulated using high molecular weight polymers to increase viscosity or salts to increase conductivity [38]. Therefore, it is possible to counteract this size effect if desired. The use of acetic acid to solubilise insulin caused a decrease in surface pH to approximately 6, which is only slightly below the natural range for human saliva and so likely to be tolerable.

Insulin was released more slowly from the fibres in comparison to the rapid release observed for highly water-soluble proteins [14, 33, 34]. A similar electrospun material comprised of a blend of a water soluble polymer (poly(vinyl alcohol)) and a gel-forming polyionic polymer (sodium alginate) containing insulin was investigated by Sharma et al. These fibres also displayed slow insulin release, with complete liberation reached only after 10 h [17]. The authors hypothesised that the slow release was mediated by the biodegradation of sodium alginate. Considering the similar release profile observed for our system, it is possible that the slow release is caused by the intrinsically slow dissolution of insulin at physiological pH. A more rapid release profile may be desirable for oral mucosal insulin delivery, as eating and drinking could dislodge the membrane and overnight application may present a choking hazard.

There is potential to increase the release rate using off-the-shelf excipients, such as basic amino acids or ethylenediaminetetraacetic acid, which are known to increase the solubility of insulin [39]. Insulin release has been investigated from several polymer-based drug delivery systems. Moreover, it is a less promising candidate for transmucosal delivery using electrospun fibres, as the low density of fibrous materials means that only relatively small masses of drug can feasibly be delivered, whereas doses of several milligrams of insulin would likely be required for sufficient absorption to achieve the desired therapeutic effect [37]. For these reasons, further investigation into the pharmacological activity of insulin upon release was not performed as part of this study.

Bradykinin causes a rapid but transient increase in intracellular calcium levels in target cells upon binding to its cell surface receptor [40], primarily through release from intracellular stores [41]. This effect that can be measured using commercially available fluorescent calcium probes. Here, we show that membrane–eluted bradykinin induced intercellular calcium mobilisation in oral fibroblasts both using flow cytometry and confocal microscopy. These results clearly indicate bradykinin released from the membrane maintained pharmacological activity. We are aware of only one other study verifying the activity of a therapeutically relevant peptide following release from an electrospun mucoadhesive membrane. Sharma et al., applied fibrous membranes containing insulin to rat sublingual mucosa and upon application, these caused a gradual decrease in blood glucose concentration, indicating successful delivery of biologically active insulin [17]. In conclusion, our mucoadhesive membrane system is suitable for the release of biologically active small hydrophilic peptides over appropriate timescales required for therapy. It constitutes an attractive new technology for the targeted delivery of permeating peptides to the buccal or sublingual oral mucosa. Potential applications include the topical release of peptides to treat oromucosal diseases locally, or alternatively transmucosal systemic delivery using an optimised dosing regimen.

## Data Availability

The datasets generated during and/or analysed during the current study are available from the corresponding author on reasonable request.
